# Identification of urinary exosomal noncoding RNAs as novel biomarkers in chronic kidney disease

**DOI:** 10.1261/rna.058834.116

**Published:** 2017-02

**Authors:** Rimpi Khurana, Glory Ranches, Simon Schafferer, Melanie Lukasser, Michael Rudnicki, Gert Mayer, Alexander Hüttenhofer

**Affiliations:** 1 Division of Genomics and RNomics, Biocenter, Medical University Innsbruck, 6020 Innsbruck, Austria; 2 Department of Internal Medicine IV, Nephrology and Hypertension, Medical University Innsbruck, 6020 Innsbruck, Austria; 3 i-med GenomeSeq Core, 6020 Innsbruck, Austria

**Keywords:** chronic kidney disease, urinary exosomes, ncRNAs, miRNAs, tRNAs, tRFs, lincRNAs, sequencing

## Abstract

In chronic kidney disease (CKD), the decline in the glomerular filtration rate is associated with increased morbidity and mortality and thus poses a major challenge for healthcare systems. While the contribution of tissue-derived miRNAs and mRNAs to CKD progression has been extensively studied, little is known about the role of urinary exosomes and their association with CKD. Exosomes are small, membrane-derived endocytic vesicles that contribute to cell-to-cell communication and are present in various body fluids, such as blood or urine. Next-generation sequencing approaches have revealed that exosomes are enriched in noncoding RNAs and thus exhibit great potential for sensitive nucleic acid biomarkers in various human diseases. Therefore, in this study we aimed to identify urinary exosomal ncRNAs as novel biomarkers for diagnosis of CKD. Since up to now most approaches have focused on the class of miRNAs, we extended our analysis to several other noncoding RNA classes, such as tRNAs, tRNA fragments (tRFs), mitochondrial tRNAs, or lincRNAs. For their computational identification from RNA-seq data, we developed a novel computational pipeline, designated as ncRNASeqScan. By these analyses, in CKD patients we identified 30 differentially expressed ncRNAs, derived from urinary exosomes, as suitable biomarkers for early diagnosis. Thereby, miRNA-181a appeared as the most robust and stable potential biomarker, being significantly decreased by about 200-fold in exosomes of CKD patients compared to healthy controls. Using a cell culture system for CKD indicated that urinary exosomes might indeed originate from renal proximal tubular epithelial cells.

## INTRODUCTION

**FIGURE 1.**
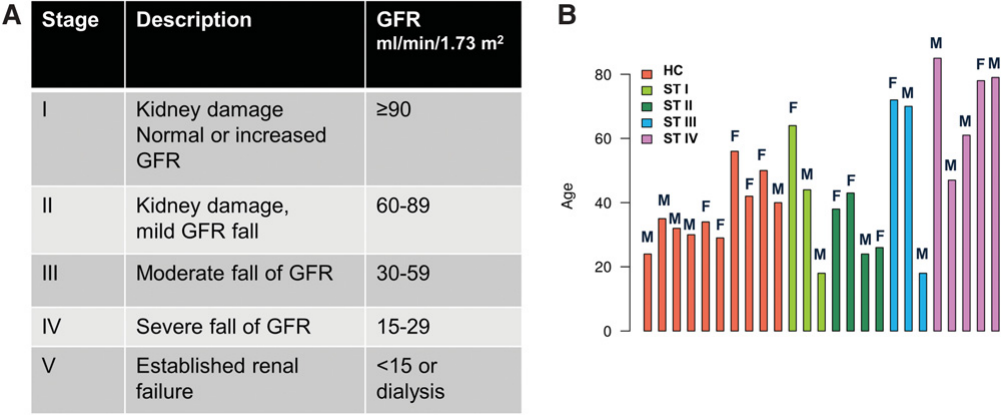
Classification of CKD. (*A*) The table represents the five stages of chronic kidney disease as assessed by analysis of the glomerular filtration rate (GFR). (*B*) The histogram displays the age distribution of healthy controls and CKD stages I–IV, including the number of females and males.

Chronic kidney disease (CKD) is defined by a reduction of the glomerular filtration rate (mostly expressed as estimated GFR or eGFR) and/or the presence of urine abnormalities, such as albuminuria, which last for longer than 3 mo (Andrassy 2013). Usually, an eGFR above 60 mL/min/1.73m^2^, without any other signs of renal pathology, is classified as normal renal function, while it is defined as stage I or II when albuminuria (>30 mg/d), hematuria, or other pathologies are present. Stage III of CKD is defined by an eGFR of 30–59 mL/min/1.73 m^2^, stage IV by an eGFR of 15–29 mL/min/1.73 m^2^, and stage V by an eGFR below 15 mL/min/1.73 m^2^ irrespective of other abnormalities present (see Fig. 1A). Using this definition, it is estimated that approximately 11%–12% of the general population suffers from CKD. Progression of CKD, i.e., a decline of the eGFR or rise in albuminuria, is associated with increased (mostly cardiovascular) morbidity and mortality, a reduced quality of life, and represents a major challenge for healthcare systems, particular when end-stage renal disease occurs and with the need of renal replacement therapy (Eckardt et al. 2013).

Clinical features associated with a worse CKD prognosis include reduced estimated glomerular filtration rate (eGFR) and increased urinary protein and albumin excretion. Histological hallmarks are an increased degree of tubulo-interstitial atrophy and fibrosis. These pathological changes are preceded and promoted by events such as infiltration by inflammatory cells, fibroblast activation and proliferation, excessive production and deposition of extracellular matrix components, and rarefaction of peritubular capillaries (Liu 2011; Mayer 2011). At the molecular level, these processes are regulated by integrated actions of various damaging as well as protective/regenerative biological pathways (Keller et al. 2012). Consequently, “omics” studies have substantially contributed to our understanding of renal disease, even though it has been realized that its transcriptional regulation is rather complex (Yasuda et al. 2006; Keller et al. 2012). One problem with studies using tissue-derived material is the fact that a renal biopsy carries a substantial risk. Thus, there is a high clinical need for methods that use other sources such as blood or urine that can be obtained more easily.

Exosomes are characterized as 30- to 150-nm diameter membrane vesicles, forming unique membrane patterns (Guescini et al. 2010), which are discharged by cells into diverse biofluids (Pan et al. 1985; Cocucci et al. 2009). They are formed within so-called multivesicular bodies (MVBs), and are secreted from cells by fusion of MVBs with the plasma membrane. Exosomes have been found in various body fluids, such as blood or urine (Théry et al. 2009; Huotari and Helenius 2011). Besides cellular plasma, lipids, and proteins, exosomes also contain various nucleic acid species including mRNAs, miRNA, lincRNAs, rRNAs, or genomic DNA (Valadi et al. 2007; Skog et al. 2008; Guescini et al. 2010; Miranda et al. 2010), which are protected from digestion by nucleases through the vesicle membrane; thereby they might be more stable compared to protein- or exosome-devoid RNAs or DNAs. Recent studies have suggested that some cellular ncRNAs are processed into smaller RNA fragments, e.g., tRNAs which are cleaved into so-called tRFs (i.e., tRNA fragments) (Thompson et al. 2008) and which were shown to be present in exosomes secreted from human semen (Vojtech et al. 2014).

Hence, we examined ncRNAs from individuals with CKD from urinary exosomes and compared them to healthy controls. In order to identify suitable ncRNA biomarkers, urinary exosomal RNAs of healthy controls and CKD patients from four different stages (I, II, III, and IV, see above) were independently isolated, reverse transcribed into cDNA and subjected to next-generation sequencing (NGS) analysis. For computational analysis of ncRNAs from NGS data, we developed a novel optimized bioinformatical pipeline, designated as ncRNASeqScan, adapted for the simultaneous identification of various classes of ncRNAs, including miRNAs, snoRNAs, lincRNAs, as well as tRNAs. We identified a significant number of novel, differentially expressed ncRNAs in CKD patients compared to healthy subjects, which might be used as diagnostic markers in CKD in the future.

## RESULTS AND DISCUSSION

### Identification of exosomal ncRNAs (exRNAs) from urine by a novel computational pipeline ncRNASeqScan

Urinary exosomes were isolated from 25 samples, including healthy controls and patients, reflecting four different stages of CKD (I, II, III, or IV, see above). CKD staging was based on classification by the glomerular filtration rate (GFR) as depicted in Figure 1A (and: see above). Urine samples were obtained after overnight fasting. The CKD group included 15 CKD patients, seven from early stages (stage I and II), eight from later stages (III and IV); healthy controls consisted of 10 samples. Patients and healthy control groups included in this study consisted of 14 males and 11 females within an age range from 20 to 85 yr (Fig. 1B). From each urine sample, exosomes were isolated by ultracentrifugation, as described. Preparation of exosomes was assessed by electron microscopy and by using exosomal markers Alix and tumor susceptibility gene-101 (Tsg101) (Théry et al. 2001; Buschow et al. 2005) (data not shown). Subsequently, total exRNA isolated from exosomes was used for cDNA library preparation and sequenced on an Ion Proton sequencer. Previous studies have indicated that most of the ncRNAs derived from exosomes exhibit a size range of 20–200 nt (Bellingham et al. 2012).

While up to now, most exRNA analyses have focused on the class of miRNAs (Cheng et al. 2014), we extended our study to also identify additional ncRNA classes from urine as diagnostic markers for CKD. Although several tools have been developed to computationally identify various ncRNA classes from NGS data, including miRdeep2 (Friedländer et al. 2012), snoSeekerNGS (Yang et al. 2010; Zheng et al. 2016), or DARIO (Fasold et al. 2011), which focus on microRNAs, snoRNAs or tRNAs, respectively, these algorithms can only identify known classes of noncoding RNA species.

In addition to known computational pipelines, a novel algorithm has previously been developed by our group, which also predicts novel ncRNA species, i.e., the automated pipeline for analysis of RNA transcripts (APART) (Zywicki et al. 2012), based on prediction of stable transcripts from RNA-seq data. In order to identify novel, as well as known differentially expressed exRNAs, we thus have combined several of these specialized tools and adapted the APART algorithm to generate a simple customizable pipeline, designated as ncRNASeqScan, which is mainly written in the R programming language. This pipeline is specialized for identification of small ncRNA species from NGS data and may be adapted for specific cases by the respective user. The toolkit provides an interface to common command line tools for adapter trimming, quality checking, mapping, RNA expression quantification, and proper genomic annotation. It also provides the possibility for analysis of relative abundance of ncRNAs and automated reporting. We thus applied the ncRNASeqScan pipeline to ncRNA transcripts, derived from exosomes, comparing the expression profiles of CKD patients to healthy controls.

### Differential abundance of exRNAs in urine of CKD patients versus healthy controls

**FIGURE 2.**
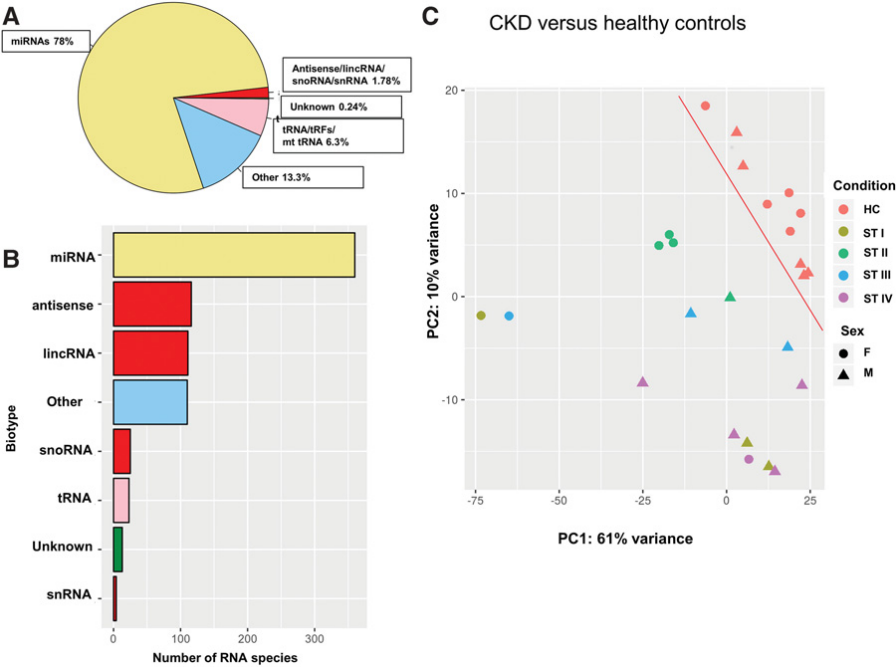
Mapped noncoding RNA identified by RNA-seq. (*A*) Percentage of total number of reads mapped to noncoding RNAs. (*B*) Number of mapped unique exRNAs from different RNA species. (*C*) PCA plot showing a clear separation between CKD patients and healthy controls (HC). Each dot represents a sample, with different colors depicting the biological group to which each sample belongs.

To investigate the abundance of exRNAs from urinary exosomes, we analyzed 15 CKD samples, including stages I–IV (see above), and compared them to 10 healthy control samples. Notably, RNA-sequencing was performed separately for CKD and healthy control groups. By using the ncRNASeqScan algorithm, we were able to detect various ncRNA classes, present in urinary exosomes from CKD patients as well as healthy controls. Collectively, in all 25 samples we identified 360 different microRNAs, which mapped to ∼78% of the reads, followed by 49 different tRNA species, which mapped to ∼6.3% of the reads, followed by antisense RNAs (116 species), lincRNA (111 species), snoRNAs (25 species), and snRNAs (4 species), respectively, which mapped to ∼1.7% of the reads (Fig. 2A,B).

**FIGURE 3.**
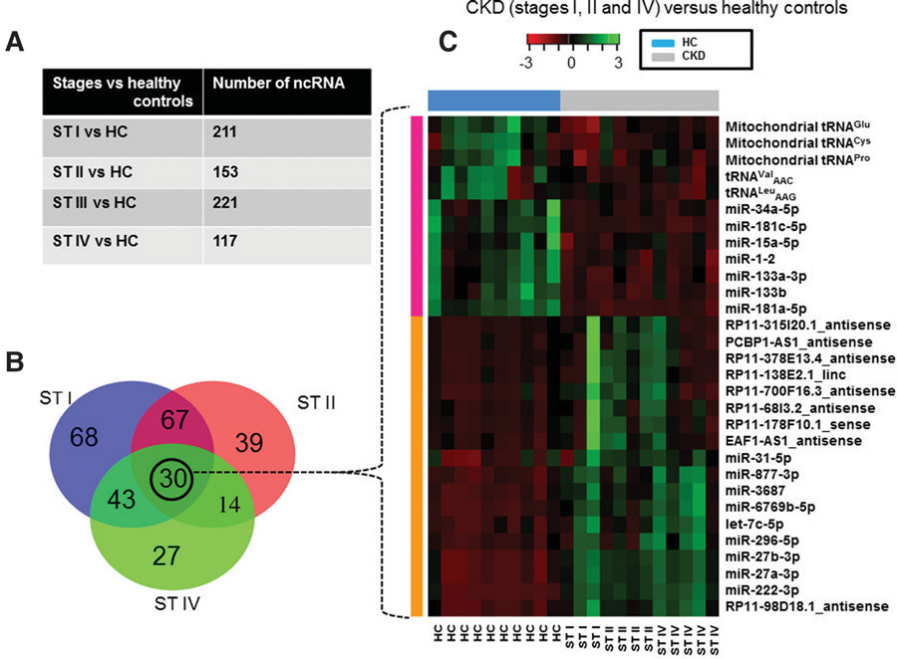
Differential abundance of ncRNA in exosomes of CKD. (*A*) The table represents the abundance of significant differences in the abundance of exosomal ncRNAs in CKD stages (ST) I–IV versus healthy controls (HC). (*B*) Venn diagram depicting the 30 overlapping differentially abundant exosomal ncRNAs between stages I, II, and IV of CKD and healthy controls. (*C*) Heatmap representing the relative abundance of 30 exosomal ncRNAs from stages I (*n* = 3), II (*n* = 4), IV (*n* = 5), and HC (*n* = 10). The color key indicates the expression change from negative (red) to positive (green). Rows represent the cluster of high abundance (orange) and reduced abundance (pink) of exosomal ncRNAs in CKD versus healthy controls.

Initially, clustering was performed on each sample according to its expression profile. We compared CKD patients to healthy control samples and we were able to separate diseased stages from the healthy control group, as depicted by the PCA plot (Fig. 2C). Although there might be a gender or age effect among CKD patients, the current sample size (i.e., 25 samples) is too small to investigate significant differences between males and females. Thus, our analysis revealed 211 ncRNAs showing significant differences in their exosomal abundance (*P-*adj <0.1) in CKD stage I, 153 ncRNAs in stage II, 221 ncRNAs in stage III, and 117 ncRNAs in stage IV, compared to healthy controls (Fig. 3A; Supplemental Fig. S2A–D; Supplemental Tables 3–6).

Upon investigation of the early stages (i.e., stages I and II) to identify early markers for CKD, 100 ncRNAs showed significant differences in their exosomal abundance between CKD patients and healthy controls. The late stages of CKD showed an overlap of 67 ncRNAs in stages III, IV, and healthy controls (Supplemental Fig. S3A). A total of 27 ncRNAs was found to be differently abundant (*P*-adj <0.1) in all the CKD stages, compared to healthy controls (Supplemental Fig. S3B). It is noteworthy that the differential abundance of exosomal ncRNAs is highly similar in all stages, implying that the majority of these ncRNAs might be used as diagnostic markers for CKD in general (Supplemental Fig. S3C; Supplemental Table 7).

Upon investigation of the PCA plot, it is evident that CKD patients from stage III cluster with all other stages. Since we were predominantly interested in identification of ncRNA biomarkers for early detection of CKD, we subsequently analyzed the differential abundance of exRNAs within stages I, II, and IV and compared them to healthy controls. In total, 30 differentially expressed ncRNA species were identified, including miRNAs, tRFS, mitochondrial tRNAs as well as lincRNAs (Fig. 3B).

### Differential abundance of exosomal miRNAs in CKD patients versus healthy controls

In these analyses, we identified 16 miRNAs showing significant differences in their abundance, with nine being significantly increased (let-7c-5p, miR-222–3p, miR-27a-3p, miR-27b-3p, miR-296-5p, miR-31-5p, miR-3687, miR-6769b-5p, and miR-877-3p) and seven being significantly decreased (miR-133a, miR-133b, miR-15a-5p, miR-181a-5p, miR-34a-5p, miR-181c-5p, and miR1-2) in CKD patients compared to healthy controls (Supplemental Tables 1, 2). Previously, it has been reported that miRNAs are the most abundant exosomal small RNA species in human urine (Lv et al. 2013).

Especially prominent, we identified miR-181a, whose exosomal abundance was significantly decreased by a factor of about 200-fold in CKD patients, compared to healthy controls in all four stages (Fig. 3C). It has previously been reported in the serum of patients with nephrotic syndrome that miR-181a might represent a potential biomarker (Sui et al. 2014a) and also might be used as an early diagnostic marker in kidney transplantation (Sui et al. 2014b). Based on these recent studies it is important to note, however, that expression of miR-181a was found up-regulated in the serum of patients with nephrotic syndrome and end-stage renal kidney disease, whereas in our analyses from all stages of CKD patients, the exosomal abundance of miR-181a was found to be significantly decreased (Supplemental Fig. S4).

Despite numerous studies, the biological functions of miRNAs, present in exosomes, still largely remain elusive. For some miRNAs, e.g., let-7c, it has been shown that transfection of let-7c combined with 5-fluorouracil (5-FU) treatment in renal cell carcinoma inhibits cell proliferation and enhances the anti-tumor efficacy of 5-FU (Peng et al. 2015). However, the function of differentially expressed urinary-derived exosomal miRNAs poses the question about their biological roles, if any. Thereby, it is conceivable that the significant increase of miR-181a in healthy controls, compared to CKD patients, could be due to an increased export of miR-181a into exosomes from healthy kidney cells or, alternatively, to down-regulation of miR-181a expression in kidney cells of CKD patients, both of which would result in decreased abundance of miR-181a in exosomes of CKD patients. This would imply, however, that exosomes are indeed secreted from kidney cells (e.g., renal tubular cells), which has not unambiguously been demonstrated, up to now (see below).

A recent study on the biological relevance of miRNAs in acute kidney injury has been observed for miR-296 using a rat model of ischemia–reperfusion injury (IRI). In addition, it has been demonstrated that expression of miR-296 is enhanced in microvesicles, arising from endothelial progenitor cells (EPCs) in hypoxic tissues; it was also shown that these microvesicles are recruited in peritubular capillaries and tubular cells, which protect the cells by enhancing tubular cell proliferation, reducing apoptosis, and infiltrating leukocytes (Cantaluppi et al. 2012).

In addition, it has been reported that let-7c is significantly down-regulated in renal tumors compared to normal tissues (Peng et al. 2015), while we observed a significant increase in their abundance in exosomes of CKD patients. Similarly, expression of miR-31 was reported to be down-regulated in polycystic kidney disease (Pandey et al. 2008), whereas in exosomes from CKD patients we found it to be significantly increased. Also, miR-222 has been demonstrated to be up-regulated in exosomes from melanoma cells, promoting tumorigenesis by activating the PI3K/AKT pathway (Felicetti et al. 2016). MiR-877 is significantly down-regulated in blood and renal cell carcinoma (RCC) tissues and is considered a potential biomarker for RCC diagnosis (Shi et al. 2016).

In summary, expression of five miRNAs has been reported to be significantly decreased in tissues related to kidney diseases as well as renal cancer (i.e., hsa-miR-296-5p, hsa-let-7c, hsa-miR-222, hsa-miR-31-5p, and hsa-miR-877-3p), while in our analyses in several cases, we find an inverse expression of these miRNAs in the urine of CKD patients. This might be consistent with a general mechanism in which a significant decrease in the presence of miRNAs in diseased tissues might be due to an increased export into exosomes (see below).

### Differential abundance of exosomal antisense RNAs in CKD patients versus healthy controls

In addition to miRNAs, eight antisense RNAs (i.e., EAF1-AS1, PCBP1-AS1, RP11-315I20.1, RP11-378E13.4, RP11-68I3.2, RP11-700F16.3, RP11-98D18.1, and RP11-1382.1; Fig. 3C; Supplemental Fig. S3C; Supplemental Tables 1, 2) were found to be differentially present in exosomes derived from CKD patients versus healthy controls. Seven out of the eight antisense RNAs were transcribed in opposite orientation to introns of predicted protein-coding genes; in one case, i.e., RP11-315I20.1, the antisense ncRNA is transcribed in opposite orientation to the LIX1L mRNA, reported to be involved in kidney cancer (Nakamura et al. 2015). The presence of antisense ncRNAs has not previously been reported in urinary exRNAs. Long intergenic noncoding RNAs (lincRNAs) or antisense RNAs, which are transcribed opposite to the sense strand of mRNAs or sense to hnRNAs or primary transcripts, have been shown to regulate gene expression in eukaryotes. Several antisense RNAs are currently used as potential diagnostic and prognostic markers involved in human diseases, such as various cancers (Hessels et al. 2003; Tinzl et al. 2004; Gibb et al. 2011). It has been shown that exosomes, secreted from three human colorectal cancer cell lines, contain specific antisense RNA species (Chiba et al. 2012). Abundance of antisense or lincRNAs in exosomes might influence expression of corresponding mRNAs in cells from which exosomes are derived (e.g., renal tubular cells, see above) or in cells from which they are taken up.

### Differential abundance of exosomal nuclear encoded tRNA fragments (tRFs) and mitochondrial tRNAs in CKD patients versus healthy controls

**FIGURE 4.**
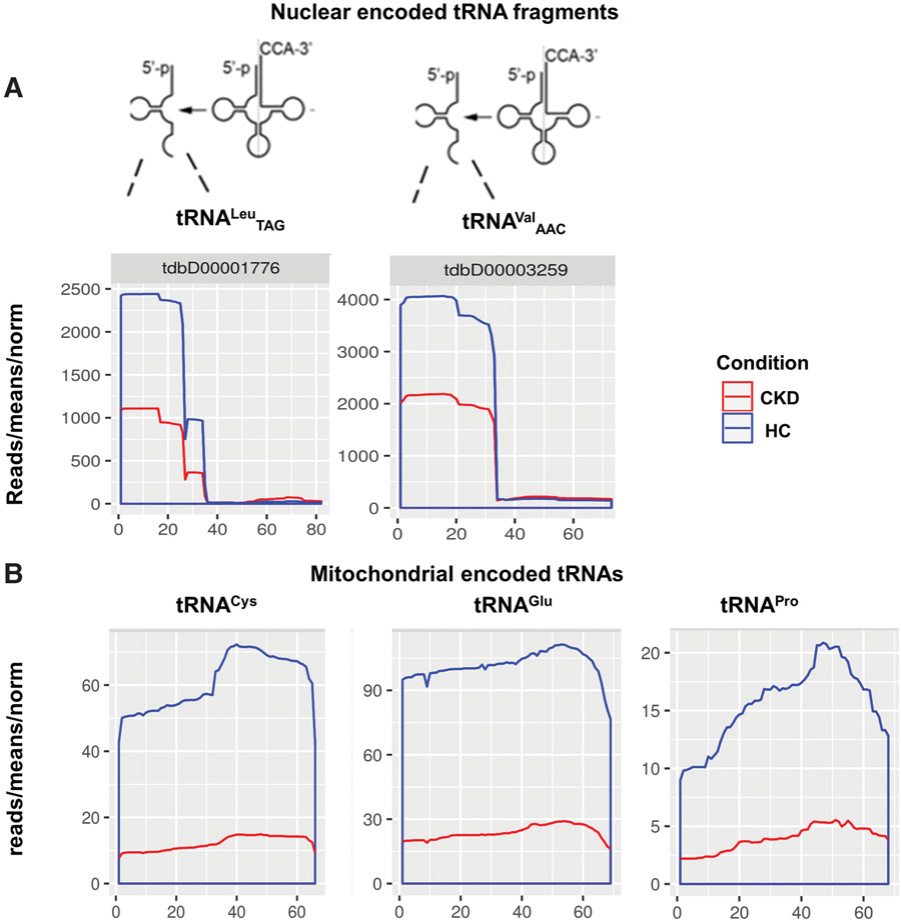
Nuclear encoded tRFs (*A*) and mitochondrial encoded tRNAs or mt-tRNAs (*B*). (*A*) The line graph represents length and number of reads of fragmented tRNAs: tRNA^Leu^ and tRNA^Val^. The graphs show the presence of fragments at the 5′ in all cases. (*X*-axis) Length of the mitochondrial tRNAs; (*y*-axis) normalized mean reads in both CKD (red) and health controls (blue). (*B*) The line graph represents length and number of reads in three mitochondria tRNAs (mt-tRNA^Cys^, mt-tRNA^Glu^, mt-tRNA^Pro^). (*X*-axis) Length of respective mitochondrial tRNAs; (*y*-axis) normalized mean reads in both CKD samples (red) and healthy controls (blue). Note that unlike in *A*, an abundance of full length tRNAs is observed.

Among differentially abundant urinary exRNAs, we also observed two nuclear encoded tRNA fragments (tRFs), i.e., tRF^Val^ and tRF^Leu^, displaying a size of about 32–36 nt (Fig. 4A). These exosomal tRFs, mapping to the 5′-ends of their corresponding full-length tRNAs, were found to be significantly decreased in their abundance in exosomes of CKD patients, compared to healthy controls.

Several studies have suggested that fragments derived from small noncoding RNAs such as tRNAs, vRNAs, rRNAs, or snoRNAs might act as a source of regulatory RNAs similar to siRNAs or miRNAs (Jochl et al. 2008; Persson et al. 2009; Haussecker et al. 2010; Falaleeva and Stamm 2013). In this context, tRFs are found in all domains of life and are cleaved into several tRF classes, based on the cleavage site. Cleavage of tRNAs in the anticodon loop divides the tRNAs into 5′ and 3′ halves (30–35 nt), while cleavage in the D-stem or T-stem results in smaller tRNA fragments sized ∼13 to 20 nt (Raina and Ibba 2014).

Interestingly, tRNA halves have previously been reported to be induced due to stress by the RNase angiogenin (Fu et al. 2009; Tuck and Tollervey 2011) and might function by down-regulation of translation due to competition with initiation factor eIF-4F, binding to eukaryal mRNAs (Svitkin et al. 2005). A second study reported that, alternatively, tRFs might function in analogy to miRNAs, i.e., by binding to the 3′ UTR of mRNAs, inhibiting the expression of RPA1 mRNA (Maute et al. 2013). Recent reports imply that YBX1 is required for loading of various RNA species, including tRFs, into exosomes (Shurtleff et al. 2016). Some studies have reported that YBX1 binds to several RNAs like tRNA fragments and miRNAs (Blenkiron et al. 2013; Goodarzi et al. 2015; Liu et al. 2015). In the present study, we found significant changes in exosomal tRFs and observed an increase in healthy controls, compared to CKD patients, which were derived from the 5′ end of the mature tRNAs (Fig. 4A).

In addition to nuclear encoded, cytoplasmic tRFs, we also observed a significant decrease in the abundance of three exosomal mitochondrial tRNAs (mt-tRNAs) in CKD patients compared to healthy controls, e.g., tRNA^Cys^, tRNA^Pro^, and tRNA^Glu^ (Fig. 4B; Supplemental Table 2). Mitochondrial tRNAs are sized about 60–68 nt. Previously, several studies have reported the presence of mt-tRNAs as well as mitochondrial proteins in exosomes (de Jong et al. 2012; Nolte-’t Hoen et al. 2012) and it has previously been demonstrated that mitochondrial proteins or RNAs are transferred by cellular vesicles between mitochondria and lysosomes (Soubannier et al. 2012). Further studies, however, will be required to elucidate whether the higher abundance of tRFs or mt-tRNAs in exosomes of healthy controls reflects a process to efficiently remove cellular or mitochondrial waste from cells (i.e., tRFs or mt-tRNAs), a mechanism that might be negatively affected in kidney cells of CKD patients.

### Are urinary exosomal ncRNAs derived from kidney-related cell types?

Recent studies have shown that the renal proximal tubule is a primary target of kidney injury and progression of kidney disease. The proximal tubule acts as a primary sensor in response to stress conditions (e.g., obstructive, ischemic, hypoxic, oxidative, or metabolic stress), which results in cell death and formation of tubular glomeruli, and thus serves as an effector in the progression of CKD (Chevalier 2016).

To assess whether urinary exosomal ncRNAs are indeed derived from kidney-related cell types, we investigated a cell culture system of renal proximal tubular epithelial cells (RPTECs) for CKD. RPTECs are reported to mimic conditions of CKD by using oncostatin M (OSM) (Sarközi et al. 2015), transforming growth factor-β1 (TGF-β1) (Yamamoto et al. 1993; Hills et al. 2009), and interleukin-1β (IL-1β) (Vesey et al. 2002).

For proof of principle, we investigated the presence of the two nuclear encoded tRFs, described above, by Northern blotting since it has previously been reported that tRFs are indeed present in exRNA preparations (Mami and Pallet 2015) and are regulated in cellular responses to kidney injuries. Here, activation of the ribonuclease angiogenin in the kidney induces tRNA cleavage, and these tRNA cleavage products are subsequently involved in the tubular adaptation upon stress (Fu et al. 2009; Mami and Pallet 2015). To our knowledge, up to now most NGS approaches have not validated differential expression/abundance of ncRNAs by Northern blotting; for our study, we selected tRFs (i.e., tRNA^Leu^ and tRNA^Val^) for Northern blot analysis because of their high abundance and because we aimed to further corroborate processing of tRNAs into tRFs by a second method.

**FIGURE 5.**
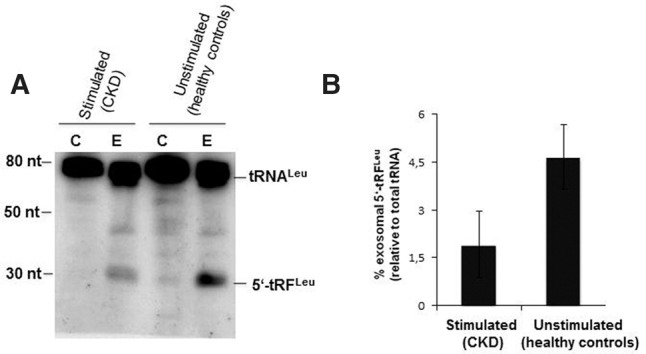
Identification of tRFs by Northern blotting. (*A*) Renal proximal tubule (RPTEC/TERT1) cells were differentiated and either left unstimulated (healthy controls) or stimulated (CKD) using IL-1β (10 ng/mL), TGF-β1 (10 ng/mL), and OSM (10 ng/mL). Total RNA was extracted from the cells (C) and supernatant, i.e., exosomes (E). (*B*) The levels of full-length tRNA^Leu^ and its derived tRF were quantified by phosphor imaging. The percentage (%) of tRF^Leu^ was compared relative to the total amounts of tRNA^Leu^ (i.e., full-length and fragment tRNA^Leu^). Bar graph represents the mean ± SD from two independent experiments.

To that end, total RNA from unstimulated and stimulated RPTEC cells and from the respective supernatant medium containing exosomes was extracted. Subsequently, total RNA from cells or exosomes was isolated and analyzed by Northern blotting, using an oligonucleotide probe targeting the 5′ ends of tRNA^Leu^ (Fig. 5) or tRNA^Val^ (data not shown). By this approach, we were able to indeed verify increased abundance of respective exosomal tRFs in unstimulated RPTECs, compared to stimulated cells, as observed in urinary exosomes of CKD patients versus healthy controls (Fig. 5).

Nevertheless, by using a cell culture system for CKD (i.e., unstimulated and stimulated RPTECs), we were able to recapitulate the relative abundance of tRFs in kidney cell-derived exosomes, as observed for urine. This might be consistent with the notion that urinary exosomes are indeed derived from kidney-related cell types, such as, for example, renal tubular epithelial cells and that their intracellular RNA composition might be mirrored by exosomes.

While our data suggest that the urinary exosomal tRF^Leu^ and tRF^Val^ may originate from kidney-related cell types, these tRFs may not exclusively be differentially expressed in CKD or renal injury, as previous studies have shown that tRFs are initiated in various pathological stress injuries, and their production is also linked to various other diseases (e.g., cancer, infection, and neurodegeneration) (Anderson and Ivanov 2014). Indeed, it has been reported that tRF levels were found elevated in the serum and urine of cancer patients (Mami and Pallet 2015). Therefore, it is plausible that these tRF candidates may also arise from other cell types in addition to kidney cells, in response to cellular stress or injury and in association with other diseases.

### Conclusion

In this study, we analyzed urinary exosomal ncRNAs by RNA-seq and generated a novel computational algorithm, designated as ncRNASeqScan, to search for ncRNAs as diagnostic markers in early CKD. Unlike for kidney biopsies, urine is an easily accessible biofluid and thus would greatly facilitate the use of biomarkers. Our novel computational pipeline supports analysis of NGS data from different platforms, i.e., Illumina (Illumina, Inc.), Ion-torrent (Ion Torrent, Thermo Fisher Scientific), or 454-pyrosequencing (Roche). NcRNASeqScan is a bioinformatics tool to enable complete analysis of comprehensive transcriptomes and identifies novel ncRNAs based on their contigs and secondary structure similarity. As compared to other pipelines, ncRNASeqScan is a novel toolkit that specializes on small ncRNA classes with the ability to handle reads that map to multiple positions in the genome, which is accomplished by a two-step mapping approach, followed by a clustering step. It features extensive annotation and provides reporting of significant differential expression analysis of ncRNAs.

By our analyses, we have identified various ncRNA classes with known or predicted functions (i.e., such as miRNAs, tRFs, or mt-tRNAs) as well as with largely unknown functions, such as antisense ncRNAs (i.e., lincRNAs), exhibiting differential abundancies in exosomes of CKD patients versus healthy controls. As for the general biological function of urinary exRNAs in CKD, two major questions have to be addressed in future experiments: (i) what is the cellular source where urinary exosomes are derived from? Applying the cell culture system of the CKD RPTEC model, we found some evidence that urinary exRNAs might indeed be derived from kidney (-related) cell types (see above); (ii) what is the function of urinary exosomes and respective urinary exRNAs? In several cases, we observed a differential abundance of specific urinary exRNAs of CKD patients, compared to healthy controls, while the same ncRNAs were reported to be inversely expressed in respective kidney-related tissues of various kidney diseases, including kidney cancer. This might argue for a “waste disposal” mechanism. Alternatively, as shown for cancer related miRNAs, some exRNAs might act as a “second messenger” to transform normal cells into cancer cells. In conclusion, by ncRNASeqScan, we investigated 15 exosomal ncRNA profiles in urine samples from CKD patients and compared them to 10 healthy controls. We identified 30 ncRNAs, comprised of miRNAs, tRNA fragments, antisense RNAs, and mt-tRNAs, being differentially abundant in exosomes of early CKD stages compared to healthy controls. Especially intriguing was an about 200-fold reduced abundance of miR-181a in CKD patients in the early as well as later stages of the disease, compared to healthy controls. Thus in the future, miR-181a as well as other identified ncRNAs might be used as biomarkers for early detection of CKD. To that end, our analysis sets the stage for applying a qPCR panel, encompassing identified differentially expressed urinary exRNAs, to a larger patient cohort for validation of suitable biomarkers in CKD.

## MATERIALS AND METHODS

### Sample collection and urine processing

Exosomes were isolated as previously described in Alvarez et al. (2012) with few modifications. Briefly, 50 mL of urine was collected from 15 patients of various stages of CKD and 10 healthy controls. CKD samples were further divided into two groups, i.e., the early stages (stage I: three samples and II: four samples) and the later stages (stage III: three samples and stage IV: five samples). To remove the urinary cell debris or cells, samples were centrifuged at 1000 rpm for 10 min, followed by 2500 rpm for 10 min at 4°C. The supernatants were transferred to Beckmann tubes and centrifuged at 187,000*g* for 1 h and 30 min at room temperature. The supernatants were saved and pellets were resuspended in 1 mL isolation solution (250 mM sucrose, 10 mM triethanolamine [pH 7.6]), followed by incubation with 50 µL 1 M dithiothreitol (DTT). Samples were vortexed vigorously, resuspended in supernatant and centrifuged again at 187,000*g* for 1 h and 30 min at room temperature. Exosomes were resuspended in 100 µL DEPC-treated water and used for total RNA extraction.

### Total RNA extraction from exosomes

DEPC-treated water was added to samples to a final volume of 400 μL. Equal amounts of Tri-reagent (Sigma) were added to each sample, and the mixture was incubated at 95°C for 5 min, cooled on ice to 4°C for 5 min, and incubated at room temperature for another 5 min. One volume of phenol chloroform isoamyl alcohol solution was added to the samples, which were centrifuged for 5 min at 13,000 rpm. The aqueous phase was carefully removed and transferred to a fresh tube. To remove residual phenol, a second chloroform extraction was carried out. Subsequently, the RNA was precipitated in two and a half volumes of ethanol and one-tenth volume of sodium acetate (3 M, pH 5.2). Samples were incubated at −80°C for 20 min and centrifuged at 13,000 rpm, 4°C, for 20 min. Pellets were washed with 70% ethanol, centrifuged at 13,000 rpm for 5 min and dissolved in 20 µL DEPC-treated water.

### RNA-sequencing analysis

For RNA-seq analysis, Ion Proton System for next generation sequencing (Ion Torrent, Life Technologies) was performed. For each sample, 10 ng of total RNA was reverse transcribed into cDNA, as described in the manufacturer's instructions. The libraries were generated with Ion Total RNA-Seq Kit v2 following the manufacturer's instructions, inhibiting cDNA synthesis of the adaptor byproducts and cDNA separation with magnetic bead-based technology. The samples are pooled and loaded on the Ion chip Kit v2 and sequenced on the Ion Proton sequencer (Ion Torrent, Life Technologies).

The Torrent Suite software (https://ioncommunity.thermofisher.com/docs/DOCS-7189) was used to filter out low quality reads and to trim 3′ adaptors, generating FASTQ file (https://en.wikipedia.org/wiki/FASTQ_format) for each sample.

### RPTEC cell culture system

Renal proximal tubule RPTEC/TERT1 cells were cultured as previously described (Limonciel et al. 2012). Briefly, cells were grown in a serum-free mixture of DMEM/F-12 (1:1) medium containing ITS (insulin [5 µg/mL]-transferrin [5 µg/mL]-selenium [5 ng/mL]), Glutamax (2 mM), EGF (10 ng/mL), hydrocortisone (36 ng/mL), and penicillin (100 U/mL)/streptomycin (100 µg/mL), at 37°C in a humidified 5% CO_2_ atmosphere. Cells were fed every 2–3 d. Following 10 d in culture, cells were incubated with growth medium containing penicillin/streptomycin only for 48 h, and subsequently stimulated with TGF-β1 (10 ng/mL), IL-1β (10 ng/mL), and OSM (10 ng/mL) for another 48 h. The supernatants and cell pellets were harvested prior to removal of supplements and after stimulation.

### RNA preparation from cells and exosomes

RNA isolation of cell pellets and exosomes was carried out using the Tri-Reagent (Sigma) and miRcury RNA isolation kit-Biofluids (Exiqon), according to the manufacturer's instructions. Samples were dissolved in DEPC-treated water and stored at −80°C. Exosomes from supernatants were isolated as described earlier.

### Northern blot analysis of tRNA^Leu^ from cells or exosomes

Total RNA, isolated from the cells or exosomes, was size-fractionated using denaturing polyacrylamide gel electrophoresis (PAGE). Each RNA sample (3 μg) was heat-denatured for 3 min at 95°C in formamide buffer and separated on an 8% polyacrylamide gel containing 7 M urea. Ethidium bromide-stained RNA was visualized by a Transilluminator (Bio-Rad) and transferred onto a Hybond N^+^ nylon membrane (GE Healthcare). The membrane was cross-linked at 120 mJ by an ultraviolet (UV) crosslinker. The oligonucleotide probe, complementary to the 5′ half of tRNA^Leu^ was radioactively labeled with [γ-^32^P]-ATP (Hartmann Analytics) using T4 polynucleotide kinase (NEB) and according to manufacturer's instructions. Following pre-hybridization, the probe was added to the membrane for hybridization at 42°C overnight. Subsequently, the membrane was washed three times with SSC (20× saline-sodium citrate: 3 M NaCl and 0.3 M sodium citrate, pH 7.0) washing buffer of different stringency (2× SSC, twice and 1× SSC, once) for 5 min each at room temperature. The radioactive signal was detected and quantified using a Typhoon PhosphorImager (GE Healthcare). The tRNA^Leu^ probe sequence used for hybridization was 5′-CCTTAGACCGCTCGGCCACGCT-3′ (Integrated DNA Technologies).

### Preprocessing mapping and assembly by ncRNASeqScan pipeline

We developed an in silico pipeline to analyze the small ncRNA transcriptome, designated as ncRNASeqScan. The pipeline focuses on mapping annotation and differential presence analysis of already known classes of ncRNAs, but also predicts transcripts based on contiguous sequencing stretches (contigs). By using this pipeline, users are able to investigate the small ncRNAs transcriptome, starting from raw sequencing reads to statistical analysis and visualization. The workflow of the pipeline is presented in Supplemental Figure S1. This pipeline is divided into seven subsections that are described as follows:

#### Preprocessing sequencing reads

In the first step, raw data are preprocessed to remove residual adapter sequences, using cutadapt (version 1.1) (Martin 2011); in the following filtration step, low quality sequences as well as sequences less than 16 nt in length were eliminated.

#### Mapping and assembly of sequencing reads to know ncRNA species

The preprocessed trimmed reads were mapped by STAR (version 2.4.0i) (Dobin et al. 2013) to a custom ncRNA genome, which contains both exonic as well as intronic sequences and was generated by using the ncRNA sequence for *Homo sapiens* (release GRCh37.75) from Ensembl (Flicek et al. 2014). Since we focused on small ncRNAs, the fasta file was restricted to contain only 400-nt-long ncRNA sequences and altered by adding mature miRNA sequences from miRbase (Griffiths-Jones et al. 2006; Kozomara and Griffiths-Jones 2014), snoRNA sequences from snoRNA-LBME-db (Lestrade and Weber 2006) and tRNA sequences from tRNAdb and tRNA predictions from GtRNAdb (Chan and Lowe 2009). The parameter set for STAR was specifically chosen for small ncRNAs and allowed multiple mapping locations up to 100 positions. The ratio of mismatches to mapped length (outFilterMismatchNoverLmax) was chosen to be less than 0.05. The minimum number of matches of a read was set to 16 and the number of multiple matches that are allowed was set to 100.

“STAR options –runThreadN 8 –outFilterMismatchNoverLmax 0.05 –outFilterMatchNmin 16 –outFilterScoreMinOverLread 0 –outFilterMatchNminOverLread 0 –alignIntronMax 1 –outFilterMultimapNmax 100 –outSAMprimaryFlag AllBestScore –outReadsUnmapped Fastx”

#### Mapping and assembly of potentially novel ncRNA species

Previously unmapped sequences were mapped in a second round to the whole genome (version hg19) from the UCSC Genome Browser (Kent et al. 2002). In this step, longer RNAs are mapped to the whole genome. The parameter set of STAR included a ratio of mismatches to mapped length (outFilterMismatchNoverLmax) of less than 0.023. The minimum number of matches of a read was set to 18 and the number of multiple matches that are allowed was set to 100.

“–runThreadN 8 –outFilterMismatchNoverReadLmax 0.023 –outFilterMatchNmin 18 –outFilterScoreMinOverLread 0 –outFilterMatchNminOverLread 0 –alignIntronMax 1 –outFilterMultimapNmax 100 –alignEndsType EndToEnd –outSAMprimaryFlag AllBestScore –outSAMtype BAM Unsorted”

In both mapping procedures, mapped sequences were sorted and indexed by using SAMtools (version 0.1.19) (Li et al. 2009). Bedgraph files for coverage analysis were compiled by utilizing genomecov from the BEDtools software (version v2.23.0) (Quinlan and Hall 2010).

#### Contig clustering

Since reads were allowed to map to multiple positions in the genome, they were clustered to a single representative locus. The clustering procedure is based on the clustering algorithm of APART (Zywicki et al. 2012) and divided into two major steps, i.e., within conditions (within healthy controls and within CKD) and between conditions (between healthy controls and CKD). Initially, contiguous sequencing stretches (contigs) are generated from sequencing reads. Thereby, mapped reads are converted from binary sequence alignment/map format (bam) to browser extendable data (bed) format to be able to count multiple read hits in the genome, by utilizing the –NH option of the BamToBed command of the BEDtools software. The resulting bed files were merged to obtain contigs that consist of a minimum number of five reads that overlap by at least 1 nt. Therefore, mergeBed of the BEDtools software in combination with an AWK script command was used.

MergeBed options -s –d -1 -c 4,5,6 -o count,mean,distinct | awk ‘{if($4 > 5) print }'

The resulting contigs were intersected by a combination of a customized perl script and the multiIntersectBed command from BEDtools. This script reports the longest possible contig, if it is present in all samples within a condition, e.g., if a contig is present in all samples of CKD patients, it is reported. Based on the resulting set of contigs, clustering is performed. All contigs that are not unique are clustered to the contig with the highest read count if they are composed of 95% of the same reads.

For each contig in the list of unclustered contigs, the contig with the highest read count is selected as representative contig (RC). If there are several contigs with the same read count, the longest contig is selected. If there are still several contigs that fulfill these conditions, one is selected at random. In the following, all reads and their mapping positions that overlap this contig are obtained. Subsequently, all other contigs that share reads with RC are reported. If these contigs share 95% of reads with the RC, they are removed from the list. Finally, the RC is added to a new list of clustered contigs and removed from the list of unclustered contigs. This process is repeated until the list of unclustered contigs is empty.

#### Annotation

Subsequently, reads were counted for each contig by using the multicov program of the BEDtools software. The potentially novel ncRNAs, represented by the resulting contigs, were subsequently annotated by utilizing the short-ncRNA-annotation package, especially the ENSEMBL annotation track and the repeat masker track from the UCSC Genome Browser. In addition, some tools specializing in mining deep-sequencing data are miRdeep (Friedländer et al. 2008) and snoSeeker (Yang et al. 2006). MiRdeep annotates known and predicts novel miRNAs. It calculates the likelihood of an alignment region based on secondary structure and assembles the sequencing reads that align to it. SnoSeeker is used to predict snoRNAs, which is based on a probabilistic model and screens sequencing data for the C/D box and H/ACA box guide and orphan snoRNAs. Inclusion of cmscan from the software Infernal (Nawrocki 2014) was utilized to annotate ncRNA candidates based on their secondary structure similarity to already known ncRNAs from Rfam (Nawrocki et al. 2015).

#### Reporting

At the end of the annotation process, NcRNASeqScan pipeline reports novel and annotated exRNAs. The resulting files include detailed sequence alignments of reads with length distribution in BAM format for ncRNAs and the hg19 human genome for each library. Moreover, the resulting annotation file consists of annotated gene information including clustered read counts with description of ncRNAs and contigs, identified positions of products and contig fasta file. Furthermore, differential presence profiling was performed using *R*.

#### Identification of differentially expressed ncRNAs

Initially, the known exosomal ncRNAs from the first mapping run were filtered by removing duplicated entries based on their annotation and complemented with the contigs from the prediction process. On the resulting set of 762 ncRNAs, differential presence analysis was performed by using the DESeq2 package with predefined parameters (Love et al. 2014). ncRNAs with an adjusted *P*-value <0.1 after multiple testing corrections as designed by Benjamini and Hochberg (1995) were considered statistically significant. The code to perform the mapping, assembly annotation, and differential presence analysis of short exosomal ncRNAs is available as an R package at https://github.com/SimonSchafferer/RNASeqUtility.

## SUPPLEMENTAL MATERIAL

Supplemental material is available for this article.

## Supplementary Material

Supplemental Material
