# Supplementary material for: Identification of urinary exosomal noncoding RNAs as novel biomarkers in chronic kidney disease
Source: RNA. 2017 Feb;23(2):142–52. doi: 10.1261/rna.058834.116 (PMC5238789; doi:10.1261/rna.058834.116)
Supplement: Supplemental Material [file supp_23_2_142__index.html]

Supplemental Material 

# Identification of urinary exosomal noncoding RNAs as novel biomarkers in chronic kidney disease

## Supplemental Material

**Files in this Data Supplement:**

- Supplemental Figure S1.tif
- Supplemental Figure S2.tif
- Supplemental Figure S3.tif
- Supplemental Figure S4.tif
- Supplemental Legends.docx
- Supplemental Table 1.tif
- Supplemental Table 2.tif
- Supplemental Table 3.xls
- Supplemental Table 4.xls
- Supplemental Table 5.xls
- Supplemental Table 6.xls
- Supplemental Table 7.xls
