# Supplementary material for: Identification of urinary exosomal noncoding RNAs as novel biomarkers in chronic kidney disease
Source: RNA. 2017 Feb;23(2):142–52. doi: 10.1261/rna.058834.116 (PMC5238789; doi:10.1261/rna.058834.116)
Supplement: Supplemental Material [file supp_058834.116_Supplemental_Legends.docx]

**Supplementary Table 1.**

The table represents fold changes in increased abundance of exosomal ncRNAs from healthy controls versus CKD patients (stage I, II and IV), as well as their adj. p-values.

**Supplementary Table 2.**

The table represents fold changes in decreased abundance of exosomal ncRNAs from healthy controls versus CKD patients (stage I, II and IV), as well as their adj. p-values.

**Supplementary Figure 1: ncRNASeqScan pipeline for ncRNAs**

Flow chart summarizing the computational pipeline for identification of ncRNA from high throughput sequencing data: The pipeline integrates several tools: Initially the raw data trimming, quality check is performed, aligned to the ncRNAs using STAR mapper, unmapped data aligned with human genome. This two-step mapping strategy is that a read, which actually originates from a non-annotated RNA, would be erroneously assigned to a similar (probably paralogous) locus and not considered in the second genome-wide mapping step. Steps for processing the contig clustering produce the final ncRNAs and annotations followed by the differential presence using R Bioconductor

**Supplementary Figure 2 Differential abundance of ncRNA in exosomes of CKD stages (stage I, II, III and IV) versus healthy controls**

Heatmaps showing the expression changes of each stage of CKD compared to healthy controls. Hierarchically clustered ncRNAs (rows), significantly differentially abundant exosomal ncRNAs between: (A) stage (ST) I (grey, n=3) and healthy controls (HC) (blue, n=10), (B) stage II (grey, n=4) and healthy controls (blue, n=10), (C) Stage III (grey, n=3) and healthy controls (blue, n=10), (D) Stage IV (grey, n=5) and controls (blue, n=10). The heatmap denotes reduced abundance in red while green denotes increased abundance.

**Supplementary Figure 3 Overlap ncRNAs between each stage**

(A) Venn diagram illustrating the number of overlapping differentially abundant exosomal ncRNAs between early and late stages of CKD: early stage: 100 overlaps in stage (ST) I and stage II (top), late stage: 67 in overlaps stage III and stage IV (centre). (B) Venn diagram showing the number of overlapping differentially abundant exosomal ncRNAs between each stage of CKD and healthy controls; 27 exosomal ncRNA overlap between in all the stages of CKD are shown. (C) Heatmap represents significantly differentially abundant exosomal ncRNAs overlapping between stages I (n=3), II (n=4), III (n=3) and IV (n=5) compared to healthy controls (n=10). The color key indicates the expression change from negative (red) to positive (green). Rows represent the cluster of up (orange) and down (pink) exosomal ncRNAs in CKD. Columns show individual group from CKD patients (stages I, II, III and IV) (blue bar) and healthy control group (grey bar). The color key indicates the expression change from negative (red) to positive (green).

**Supplementary Figure 4 Volcano plots comparing differential exosomal ncRNA abundance between healthy controls and CKD sample types at different stages**.

Each color represents ncRNA biotype. The x-axis is log2 fold change value and y-axis –log10 (p-value). The upper left corner represents the highly reduced abundant miR-181a in each stage (ST) of CKD. Each biotype is represented by separate color. The horizontal red line represents untransformed p-value of 0.01, so points above it have smaller p-values.

**Supplementary Table 3.** List of differentially abundant exosomal ncRNAs from stage I of CKD patients vs healthy controls

**Supplementary Table 4.** List of significant differential abundance of ncRNAs in stage II from CKD and healthy controls

**Supplementary Table 5.** List of significant differential abundance of ncRNAs in stage III from CKD and healthy controls

**Supplementary Table 6.** List of significant differential abundance of ncRNAs in stage IV from CKD and healthy controls

**Supplementary Table 7.** List of significant differential abundance of ncRNAs in all stages (I, II, III and IV) of CKD and healthy controls
